# A transcription factor by any other name - function beyond DNA binding and transactivation

**DOI:** 10.1038/s44319-026-00893-3

**Published:** 2026-09-10

**Authors:** Christina Maria Schuh, Joshua Mark Brickman

**Affiliations:** Novo Nordisk Foundation Center for Stem Cell Medicine (reNEW), Department of Biomedical Sciences, University of Copenhagen, Blegdamsvej 3B, Copenhagen N, 2200 Denmark

**Keywords:** Chromatin, Transcription & Genomics

## Abstract

Transcription is a highly regulated process, and transcription factors (TFs) play a central role in determining when and where transcription occurs. While TF binding is essential for transcription, recent observations shed new light on the relationship between sequence-specific DNA binding and transcription. TF occupancy frequently does not correlate with transcriptional output, and TFs can be recruited to sites where transcription is already active, blurring conventional notions of cause and effect. Moreover, the textbook view of defined domains for DNA binding and transcriptional regulation has begun to dissolve, with the roles of intrinsically disordered domains (IDRs) in both processes making it difficult to decipher how specificity and target regulation are controlled. Why are these disordered regions so common in TFs, and how do they contribute to the identity and function of these factors? In this review, we explore the nature of TFs and the consequences of their binding for cell state transitions. Where and when do they act to stimulate transcription, find their targets, and interface with signaling to propagate specific gene expression programs?

## Abstract (web summary)

This review discusses the nature and function of transcription factors - beyond the simple view of DNA recognition and transcription activation - and what kind of roles intrinsically disordered domains play.

## Graphical abstract

**Figure:**
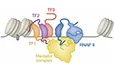

## Introduction

Transcription is the first step in regulating gene expression, shaping cellular identity and function. It is the process by which the DNA sequence is transcribed into RNA that is then exported to different regions of the cell. Transcription is carried out by RNA polymerase (RNAP), an enzyme that synthesizes complementary RNA from a DNA strand. There are three different polymerases in eukaryotes, distinct enzymes producing different types of RNAs; RNAP I and III transcribe ribosomal RNA (rRNA) and transfer RNA (tRNA), respectively, while RNAP II produces messenger RNA (mRNA) and small RNAs (Sentenac, 1985).

As mRNA is crucial for establishing cell states, its expression must be tightly and dynamically regulated to accommodate changes during development and differentiation (He et al, 2020). The engagement of RNAP II at the start site of transcription is governed by two sequence elements: the promoter, located close to the start site, and enhancers, which can be positioned at great distances upstream or downstream of the start site. Both elements are known to bind TFs and cofactors, which are both crucial for the recruitment of RNAP II to a particular gene and for regulating its activity (Andersson and Sandelin, 2020).

TFs in eukaryotes typically act in a combinatorial or cooperative manner, individually binding DNA with relatively weak affinity, and acting in concert to mediate precise changes in gene expression (Junion et al, 2012; Kribelbauer et al, 2019; Spitz and Furlong, 2012; Whyte et al, 2013). This cooperativity can be manifested either in simultaneous DNA binding or via distinct activities at multiple steps on the pathway to gene activation (Chronis et al, 2017; Hai and Curran, 1991; Junion et al, 2012; Knudsen et al, 2023; Panne et al, 2007). From the binding of TFs to enhancers, the interface of enhancer activity with promoters, and the progression of RNAP II into the gene body, TFs have been implicated in cellular competence and plasticity, the response to signaling pathways, and the putative formation of hub-like structures with environments that facilitate transcription (Chambers et al, 2003; Junion et al, 2012; Chandramohan et al, 2025; Hamilton et al, 2019; Linneberg-Agerholm et al, 2024; Niwa et al, 2000; Redó-Riveiro et al, 2024; Zamudio et al, 2019; Boija et al, 2018). What is the actual function of TFs and how multifaceted are they?

For many years, the spatial and temporal control of gene expression was thought to lie in the recognition of specific sequences in enhancers and promoters by sequence-specific DNA-binding proteins and their activation of transcription (Spitz and Furlong, 2012). However, several recent observations call the fundamental assumptions on which this dogma is founded into doubt, questioning the simple view of DNA recognition and the role of TFs in establishing transcriptional states. In this review, we discuss the function of TFs and how this can be seen in a more nuanced light based on recent observations and findings.

## Assembly of the transcription complex

To begin to understand TFs, we first consider the steps where they can regulate transcription. Promoters are located close to the transcription start site (TSS) of the gene they regulate, bind a combination of TFs, and the general transcription factors (GTFs). GTFs are ubiquitous factors required for RNAP II to transcribe DNA (Orphanides et al, 1996). These factors were initially identified through biochemical fractionation of nuclear extracts as core components required to reconstitute transcription in vitro (Matsui et al, 1980; Samuels et al, 1982). While originally defined biochemically, the identity of the proteins with in these fractions has long since been established (Buratowski et al, 1989). GTFs participate in RNAP II complex assembly, leading to the formation of a preinitiation complex (PIC) (Greber and Nogales, 2019). Following RNAP II recruitment, additional GTFs become incorporated in the PIC, stabilizing its formation via protein-protein and additional protein-DNA interactions (Greber and Nogales, 2019). The assembly of the PIC is based on in vitro reconstitution experiments, and the extent to which these fractions exist as independent entities in vivo is unclear.

The most well-studied core promoter sequence is the TATA box, which is localized ~25–35 base pairs upstream of the TSS (Goldberg, 1979; Sassone-Corsi et al, 1981). It is recognized by the TATA binding protein (TBP), which is the core subunit of TFIID (Kim et al, 1993; Burley and Roeder, 1996). The binding of TBP to the TATA box allows the recruitment of TFIIA heterodimer and TFIIB, which bind the pre-formed TBP-DNA complex and stabilize its interaction (Cortes et al, 1992; Coulombe et al, 1994; Lagrange et al, 1996; Nikolov et al, 1995). In addition to TBP, other peptides contained in the original TFIID fraction, or TBP-associated factors (TAFs), are also thought to enhance complex assembly by interacting with core promoter elements (Kadonaga, 2012). While TATA-containing promoters were the original paradigm for PIC assembly, a large subset (~76%) of human promoters lacks a TATA motif (Yang et al, 2007). At these elements, the TFIID complex still nucleates PIC formation, but different TAFs recognize elements such as the Initiator (Inr) or G-rich sequences downstream of the TSS, called distal sequence elements (DSE) (Kaufmann and Smale, 1994; Ngoc et al, 2017; Santana et al, 2022). PIC assembly occurs in a stepwise manner, beginning with TFIID binding at the promoter, followed by the addition of TFIIA and TFIIB, RNAP II with TFIIF, and finally TFIIE and TFIIH (Farnung and Vos, 2022; Orphanides et al, 1996). Within TFIIH, the XPB subunit acts as a DNA translocase, promoting DNA unwinding as a consequence of moving the DNA helix downstream to generate torsional stress that results in the formation of the transcription bubble. The CDK7 kinase subunit of TFIIH phosphorylates serine 5 within the carboxy-terminal domain (CTD) of RNAP II and thereby facilitates the initial phase of RNA synthesis (Phatnani and Greenleaf, 2006; Shiekhattar et al, 1995). The recruitment and enzymatic functions of GTFs provide multiple opportunities for regulation.

## Transcription factors

In the following section, we consider how TFs have been defined historically, their structural features, and related activities.

### DNA-binding domains

**Figure 1:**
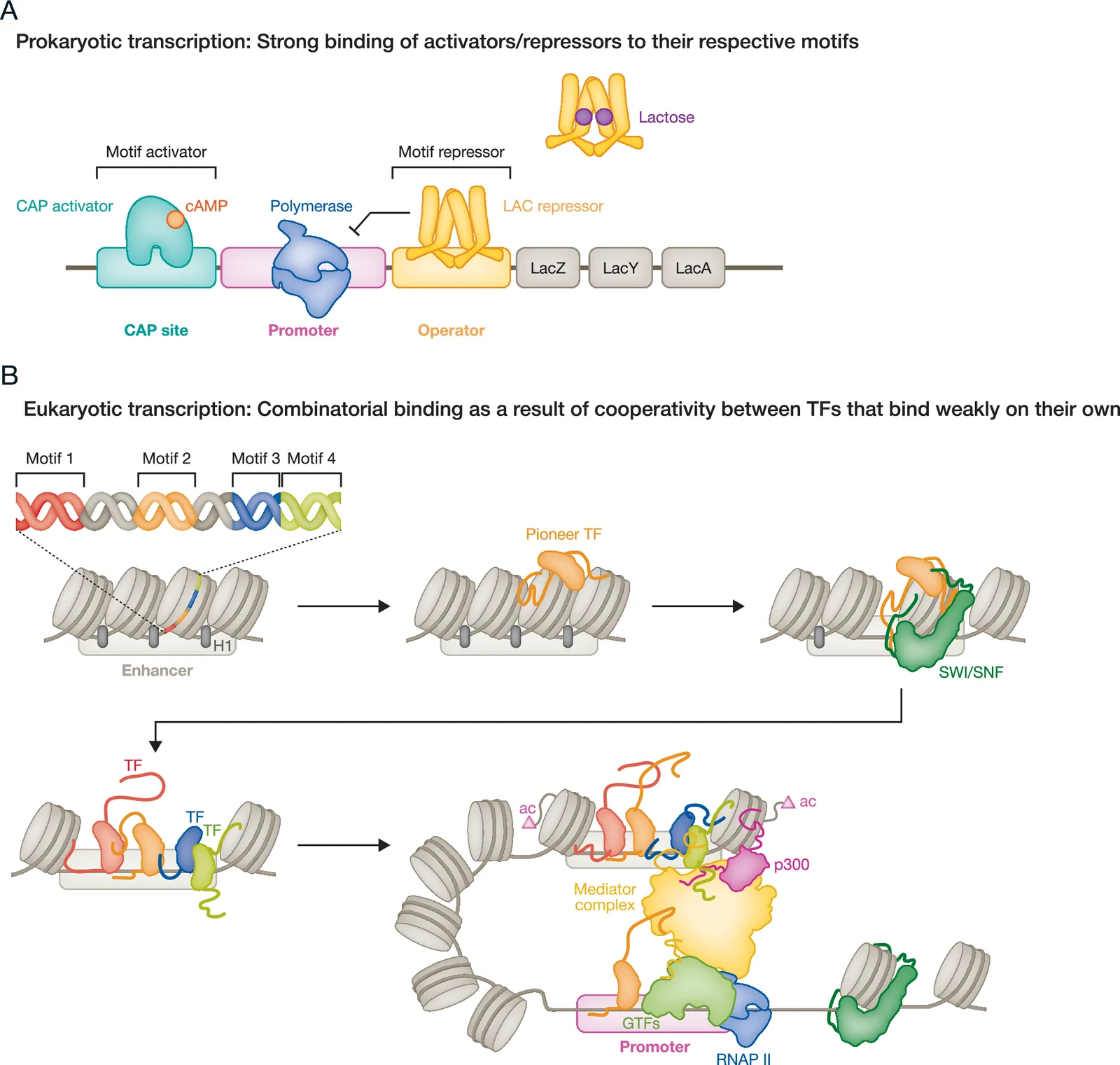
Regulation of transcription in prokaryotes versus eukaryotes. Prokaryotic TFs bind DNA with high affinity, enabling them to act independently as activators or repressors. In eukaryotes, the lower affinity of TFs necessitates combinatorial interactions, either via TF–TF, TF–cofactor, or TF–chromatin interactions. (**A**) The *lac* operon is regulated both by a repressor (LAC repressor binding the *lac* operator) or an activator, catabolite activator protein (CAP at the CAP binding site). The LAC repressor binds to the operator in the absence of lactose and prevents the polymerase from transcribing the genes in this cluster. If lactose is present, it binds the LAC repressor and inhibits its DNA binding, enabling polymerase to  transcribe the operon. Under high glucose conditions, CAP associates with cAMP, stimulating its recognition of the CAP site, allowing the recruitment of polymerase. Under low glucose conditions, cAMP fails to bind CAP, and CAP can no longer bind DNA. (**B**) Eukaryotic regulatory elements such as enhancers carry multiple motifs to bind different TFs, which act in a cooperative manner  to induce transcriptional events at target promoters. Eukaryotic TFs bind weakly to their motifs, but through their interactions with other TFs and cofactors, they can create a robust transcriptional response. Pioneer factors can bind DNA in closed chromatin and cooperate with chromatin remodelers (e.g., SWI/SNF) to open chromatin and allow other TFs to recognize their motifs. To activate transcription, TFs are thought to recruit cofactors such as, histone acetylases (e.g., p300), the Mediator complex, GTFs and RNAP II.

TFs recognize specific DNA elements via their DNA-binding domain (DBD), a structural unit that presents a unique set of amino acids in positions that can sit in the groove of the DNA helix, forming hydrogen bonds with specific DNA bases or the underlying phosphate backbone (Kribelbauer et al, 2019). The sum of these interactions determines the affinity of a TF for a specific DNA sequence. In prokaryotes, TFs typically bind DNA with extremely high affinity, often to highly specific sites with dissociation constants around 10⁻¹² M (Gilbert and Müller-Hill, 1967) (Fig. 1A). In contrast, most TFs in multicellular eukaryotes recognize shorter sequences with much lower affinity (Kribelbauer et al, 2019; Scaffidi and Bianchi, 2001). Eukaryotic TFs, therefore, require combinatorial interactions to achieve similar levels of specificity, enabling a single TF to regulate a diverse set of multicellular behaviors, depending on the different interaction partners (Spitz and Furlong, 2012) (Fig. 1B). Thus, a typical eukaryotic regulatory element can integrate multiple layers of information before embarking on a lineage-specific transcriptional program (Chambers and Tomlinson, 2009; Chronis et al, 2017; Junion et al, 2012).

In eukaryotes, there are a number of distinct evolutionarily related families of DBDs, each characterized by its own structural motif. The most common classes of TFs include zinc finger proteins (ZFPs), nuclear hormone receptors (NR), High Mobility Group domain (HMG) containing proteins, the homeodomain factors, the basic helix-loop-helix (bHLH) and basic leucine zipper (bZIP) proteins (Johnson and McKnight, 1989). Residues within these domains mediate interactions in the major groove of DNA (Ogata et al, 1994), interactions with and opening of the minor groove (Love et al, 1995), and dimerization (Kerppola and Curran, 1991). Through these interactions, DBDs are thought to confer precise sequence-recognition.

Studies of in vitro binding suggest that these functional units are sufficient to recognize specific DNA sequences, supporting the idea that DBDs determine TF specificity. This work included biochemical approaches such as DNAse footprinting (Dynan and Tjian, 1983), electrophoretic mobility shift assays (EMSA) (Fried and Crothers, 1981) and Systematic Evolution of Ligands by Exponential Enrichment (SELEX) (Jolma et al, 2013) to identify specific DNA sequences that are bound by TFs, alongside structural studies based on X-ray crystallography (McKay and Steitz, 1981), Cryogenic Electron Microscopy and nuclear magnetic resonance (NMR) spectroscopy (Gadea and Nikolova, 2023). The conserved characteristic structures of DBD families enable the prediction of unknown TF–DNA interactions using computational approaches based on Hidden Markov Models (Finn et al, 2016; Kummerfeld and Teichmann, 2006) and deep machine learning tools (Kim et al, 2021; Liebold et al, 2025).

While these in vitro approaches have been useful, they only partially predict TF binding in vivo. Whole genome approaches, such as chromatin immunoprecipitation followed by sequencing (ChIP-seq), cleavage under targets and release using nuclease (CUT&RUN), or chromatin endonuclease cleavage-sequencing (ChEC-seq), enable the mapping of TF binding in vivo, and identify the DNA sequences they recognize, typically represented as a DNA motif (Johnson et al, 2007; Skene and Henikoff, 2017; Zentner et al, 2015). Comparisons of in vitro consensus sites and in vivo binding patterns suggest that TFs interact with a wider range of sequences than expected from their in vitro activity (Badis et al, 2009; Slattery et al, 2014; Wang et al, 2012; Yang et al, 2006). Even among idealized consensus sites, only a fraction of consensus sites are occupied, indicating that the DBD alone cannot fully explain binding specificity (Harbison et al, 2004; Iyer et al, 2001; Lupien et al, 2008; Ren et al, 2000; Wang et al, 2012; Yang et al, 2006). As DNA in vivo is packed into chromatin, nucleosomes or higher-order packing can block TF binding, limiting their access to the full spectrum of DNA sites within the genome (Grand et al, 2024). Consistently, TF binding is enriched in regions of open chromatin and reduced where densely packed nucleosomes are contained in heterochromatin (Becker et al, 2016; Guertin and Lis, 2013; Lupien et al, 2008). While this selective binding could reflect chromatin access, even TFs that can recognize their sites in chromatin bind only a small fraction of their full complement of high-affinity sites (King and Klose, 2017; Reiter et al, 2017; Slattery et al, 2011). Recent experiments in yeast also suggest that deletion of DBDs across a broad spectrum of different TFs impairs DNA binding, yet many TF mutants localize to the majority of their known DNA sites (Brodsky et al, 2020; Kumar et al, 2023). While this analysis only focuses on the top target promoters recognized by these factors, it uses ChEC-seq, which gives a high non-specific signal (VanBelzen et al, 2024). Similar experiments on a smaller subset of factors show that, although cooperative binding between known partners promotes accurate sequence recognition, its overall impact is modest (Brodsky et al, 2020). In contrast, removing IDRs of these proteins in yeast causes a much greater reduction in specificity than removing the DBD, suggesting that in vivo, IDRs play a direct role in TF localization (Brodsky et al, 2020; Kumar et al, 2023). While IDRs have not been shown to mediate localization in higher eukaryotes, the recruitment of TFs to sites of active transcription has been observed, and this could be IDR-dependent (Zamudio et al, 2019; Boija et al, 2018; Abidi et al, 2026). As a result, the top-bound promoters in yeast, that are shown to be bound in an IDR-dependent manner (Brodsky et al, 2020), might similarly reflect active sites of transcription. Why should TFs be recruited to sites that are already transcriptionally active? Perhaps to enhance robust transcriptional responses and sequester them away from the majority of consensus sites in the genome, where they could disrupt cellular identity (Monteiro et al, 2026). At places where cell type-specific cooperative interactions support high-affinity binding, they recognize their sites, even at a low concentration. The remainder of sequence-specific sites are targets for these factors in other cell types and lineages, but promiscuous binding to them could disrupt cellular identity.

### Effector domains (EDs)

DBDs can recognize and localize specific DNA sequences, but on their own, they are not enough to drive transcription (Brent and Ptashne, 1985). Early studies on GAL4, a TF in yeast, showed that deleting two specific regions outside the DBD abolished the ability of GAL4 to activate transcription (Keegan et al, 1986; Ma and Ptashne, 1987a). The existence of regulatory regions outside of those regions responsible for DNA binding gave rise to the concept of activation domains, protein fragments that could activate transcription when fused to a heterologous DBD bound to its target sites (Brent and Ptashne, 1985). In addition to activation domains, distinct transcriptional repressors also contain domains that are sufficient to repress transcription on a heterologous DBD (e.g., *Drosophila* engrailed, Jaynes and O’Farrell, 1991).

Different approaches to randomization have been used to define a consensus for regulatory domain composition (Abedi et al, 2001; Ma and Ptashne, 1987b; Saha et al, 1993). Initially, these approaches were applied to libraries of random DNA, suggesting ~1% of DNA sequences could activate transcription and that these were largely acidic protein sequences (Ma and Ptashne, 1987b). Similar experiments identified positively charged peptides that repress transcription (Saha et al, 1993). With the availability of modern sequencing-based approaches, these screens have been repeated at higher resolution, and these have largely confirmed the original observations (Arnold et al, 2018; Klaus et al, 2022; Mukund et al, 2023; Soto et al, 2022; Staller et al, 2018; Tycko et al, 2020).

**Figure 2:**
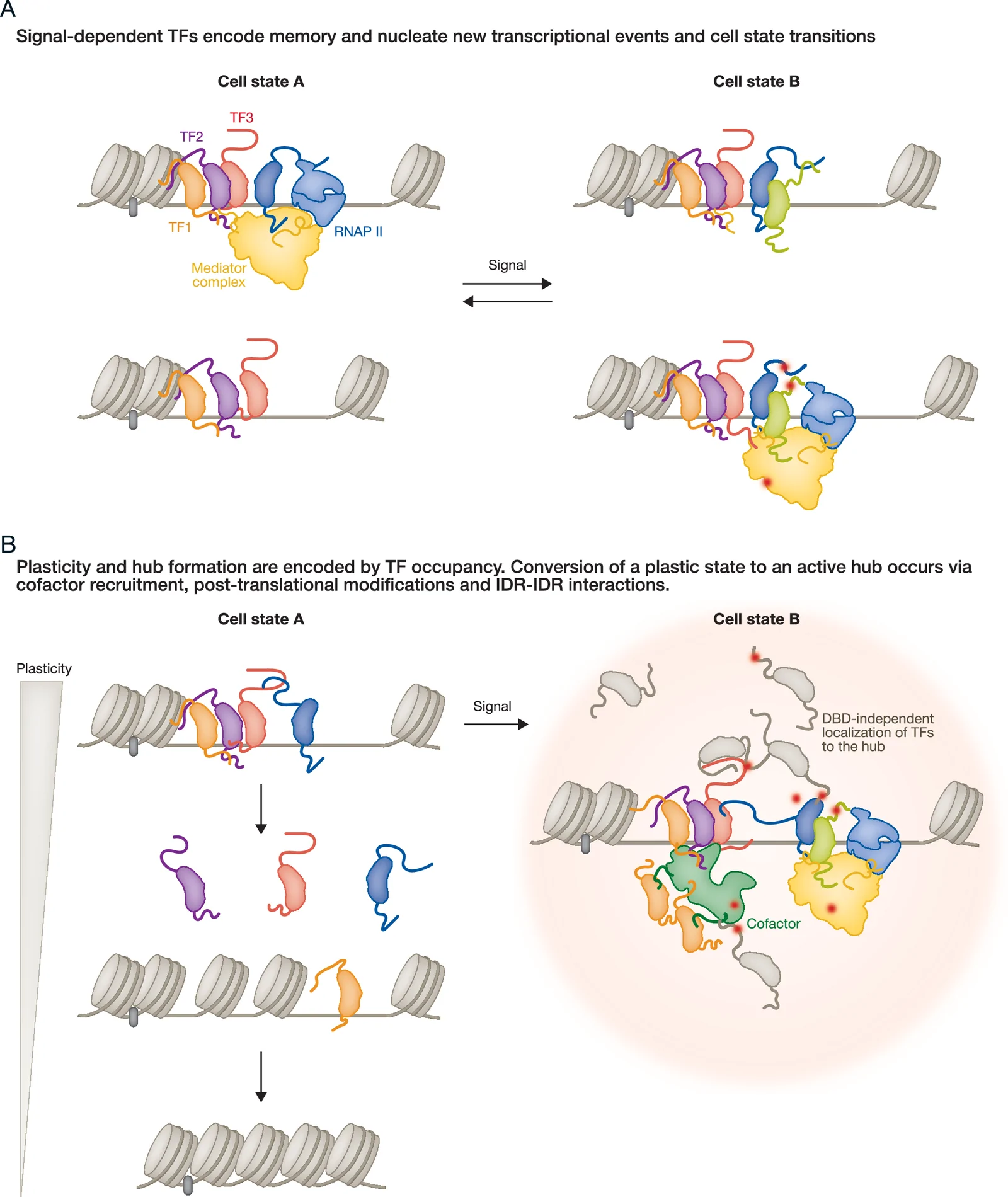
TFs acting as competence factors and as drivers of transcription (**A**) An example of how a signal nucleates new transcriptional events at enhancers to drive cell fate transitions. On the left, enhancers promoting  the endogenous gene network are active and bound by a set of different TFs to support cell state A. At the same time, a similiar set of TFs also bind enhancers, which will determine the potential cell state B once a signal has been recieved. In the latter instance, TFs are acting as competence factors for future transcriptional events (on the right). Once the signal becomes active TFs bound to enhancers permissive for the signal and/or cofactors responding to it, are modified leading to the recuitument of Mediator and RNAP II. These events lead to the activation of new transcription that promotes the transition towards cell state B. Enhancers driving cell state A are deactivated through the disengagement of Mediator and RNAP II, while TFs stay bound to serve as factors promoting competency, plasticity or cellular memory (**B**) Plasticity is dependent on steady state levels of TFs and their affinity for DNA. Memory or potential is encoded by the level of TFs present in the cell and the extent they sit at an inactive enhancer. When a signal is received by the cell the TF bound enhancer(s) are converted into hubs of transcriptional activity. Here, as above, signaling acts to recruit cofactors, additional TFs (grey), and modifies specific protein sequences, all culminating in the formation of a potential transcriptional hub characterized by a high concentration of interacting IDRs. Possible post-translational modifications are indicated by red dots.

As discussed above, activation domains were described as rich in acidic residues and showed no evidence of any structural motif (Ptashne, 1988; Sigler, 1988). These disordered domains were believed to form structure upon interacting with positively charged sites found in transcriptional cofactors (Kussie et al, 1996; Radhakrishnan et al, 1997; Staller et al, 2022). More extensive analysis of these regions demonstrates that in addition to acidic patches, they include stretches of hydrophobic residues and polyglutamine (Barbosa Pereira et al, 2023; Staller et al, 2022), all supporting the notion that they are intrinsically disordered, and hence their more recent designation as “intrinsically disordered domains,” (IDRs) (Barbosa Pereira et al, 2023; Liu et al, 2026; Staller et al, 2020, 2018). These IDRs are now thought to form multivalent interactions with multiple factors simultaneously, enabling TFs to engage with a wide range of cofactors and influence transcriptional outcomes (Brzovic et al, 2011; Currie et al, 2017; Datta et al, 2025; Herbig et al, 2010; Ozkan et al, 2025; Teufel et al, 2007). While IDRs are also involved in repression, repressors additionally contain short peptides that interface with corepressor complexes, known as short linear motifs (SLiMs) (Hu and Lazar, 1999; Klaus et al, 2022; Turner and Crossley, 1998). SLiMs are smaller than IDRs, around 3–10 aa in length, and target specific cofactors. For instance, the PxDLS motif in Snail binds to the corepressor CtBP and the WRPW motif, first characterized in Hairy factors, recruits Groucho to repress transcription (Jiménez et al, 1999; Nibu et al, 1998). Although these SLiMs, or microdomains, are not exclusively associated with repression (Næs et al, 2020; Staller et al, 2018; Warfield et al, 2014), those that are related to activation are typically enriched in acidic residues (Glu and Asp) and contain interspersed bulky hydrophobic residues such as Trp, Phe, Tyr, and/or Leu (Staller et al, 2018; Kotha and Staller, 2023). Notably, a large proportion of TFs, particularly homeodomain TFs, contain bifunctional EDs, capable of both activating and repressing transcription (DelRosso et al, 2023; Mahendrawada et al, 2025; Soto et al, 2022), although the extent to which TFs have multiple functions is controversial (Mindel and Barkai, 2025). However, in mammalian embryonic stem cell (ESC) models, TFs are frequently found as activators of stem cell genes and also directly associated with differentiation genes (Hamilton et al, 2019; Heurtier et al, 2019). At these differentiation genes, stem cell TFs do not activate transcription until a differentiation signal is received, and thus act as competence factors that support differentiation without driving it (Fig. 2A).

Given their disordered character and the importance of charge in mediating their interactions with other factors, EDs are often targets of post-translational modifications (PTMs), which can alter their properties and produce novel regulatory outcomes that are especially important in signaling-dependent transcriptional regulation (Datta et al, 2025). For instance, the ED of the AP-1 transcription factor JUN is phosphorylated by the Jun-N-terminal-kinase (JNK), which can alter transactivation and interaction with cofactors, ultimately regulating transcriptional outcome (Aguilera et al, 2011; Minden et al, 1994; Weiss et al, 2003). JNK-dependent phosphorylation alters the charge in the IDR, enabling it to manifest its activity as a transcriptional activator by promoting the dissociation of JUN from repressive complexes such as Histone deacetylase 3 (HDAC3) (Weiss et al, 2003) or Nucleosome remodeling and histone deacetylation complex (NuRD) (Aguilera et al, 2011).

### Intrinsically disordered regions

In the preceding section, we discussed the association of IDRs with regulatory domains. Yet up to 62% of TF coding sequences are contained in an IDR, in contrast to prokaryotic TFs, where this number is 5%, and for non-transcriptional eukaryotic proteins, this number is similarly lower, around 20% (Fukuchi et al, 2009; Már et al, 2023; Minezaki et al, 2006). IDRs in TFs on average span 160 aa, but are found to extend up to 600 aa, suggesting that the majority of TF protein coding sequences consists of regions of low structural complexity, without defined secondary structure (Már et al, 2023). These domains also have a high proportion of amino acids with charged or polar properties, which inhibits protein packing and maintains solvent-exposed regions that can hydrogen bond with cofactors (Sigler, 1988; Staller et al, 2018). As a result of this reduced packing, these regions are flexible and can rapidly adopt multiple conformations, allowing them to interact with a wide range of transcriptional regulators or with DNA itself (Már et al, 2023). Their positioning in EDs supports interactions with cofactors or factors of the general transcriptional machinery through “fuzzy” binding or by undergoing a disordered-to-ordered transition (Brzovic et al, 2011; Ferrie et al, 2022; Tuttle et al, 2018, 2021). Due to their chemical properties, IDRs have also been implicated in regulating liquid-liquid phase separation (LLPS) of TFs and cofactors, a phenomenon that has been linked to transcriptional hub formation (Boija et al, 2018; Ji et al, 2024; Zamudio et al, 2019; Zhao et al, 2023). For example, the IDRs of the TFs OCT4 and GCN4 or the cofactors MED1 and BRD4 have been shown to support the formation of condensates in the nucleus (Boija et al, 2018; Sabari et al, 2018). The physical properties of these condensates have been probed based on TF diffusion as measured by Fluorescence recovery after photobleaching (FRAP) and Single-particle tracking, in vitro droplet formation assays with 1,6-hexandiol and DNA Fluorescence In Situ Hybridization, but this evidence is not fully conclusive (Darzacq and Tjian, 2022; Düster et al, 2021; Stortz et al, 2024; McSwiggen et al, 2019). Even though the mechanism by which these condensates form or are related to transcription is not fully understood, those studies show that IDRs can promote the formation of biomolecular aggregates that could be permissive for transcription.

As IDRs are such a ubiquitous component of TF sequences, their role in transcription presumably goes beyond ED function. As discussed, above, IDRs have been implicated in DNA binding by TFs. In yeast, IDRs of Msn2 or Yap1 appear to mediate localization to promoters, independently of their DBD, although this study only considered the most active promoters in the cell (Kumar et al, 2023; Brodsky et al, 2020). Despite this caveat, these studies suggest that TF target search operates via a two-step mechanism: Initially, the IDR facilitates the identification of the appropriate set of promoters through multiple weak interactions with DNA or by recruitment to an active transcription complex; subsequently, the DBD recognizes and binds to the specific motif at the correct site (Brodsky et al, 2020). The ability of IDRs to influence TF binding is not unique to yeast proteins; similar findings have been reported for human hypoxia-inducible factors HIF-1α and HIF-2α that bind identical sites in vitro, but in vivo, they bind distinct target sites based on their IDRs (Chen et al, 2022). Similarly, single-particle imaging has shown that IDRs can promote the association of a TF with chromatin through interactions with IDRs of other TFs. This recruitment is independent of DBD-specificity, suggesting that IDRs engage in cooperative behavior to promote TFs to concentrate at their target sites (Abidi et al, 2026). Besides TF–TF interactions, this stabilization could also be a manifestation of negatively charged IDRs interacting with positively charged chromatin proteins like histones (Zhou et al, 2024), or a result of a regulatory interaction that IDRs have with additional transcriptional cofactors, feeding back to stabilize DNA binding. Thus, a TF does not merely bind a specific sequence and regulate transcription, but its ability to recruit cofactors and/or activate transcription can feed back on its localization, suggesting that a more nuanced approach to defining TF function in a particular context is necessary.

## Transcriptional regulatory regions and TF recognition

For TFs to regulate transcription, they generally need to recognize their respective TF binding site (TFBS), a specific DNA sequence, which in eukaryotes is between 6 and 12 bps long (Stewart et al, 2012). As discussed above, binding involves more than simple sequence recognition and often requires multiple layers of interaction. In this section, we consider how TFs locate these sites within regulatory regions, exploiting both canonical DNA binding and the potential activity of IDRs. While the transcriptional machinery assembles at promoters to initiate transcription, TFs also bind to distal regulatory elements known as enhancers, which play a critical role in controlling transcriptional activity (Furlong and Levine, 2018; Spitz and Furlong, 2012).

### Enhancers

Enhancers are regulatory regions that are located at remote locations relative to the start site of transcription. They can be found in either intragenic or intronic regions and can be located many megabases from the gene they regulate (Borsari et al, 2021; Lettice et al, 2003). Originally, they were defined as both position and orientation-independent regulatory elements, and they are essential for regulating complex patterns of gene expression in development and differentiation (Banerji et al, 1981; Lettice et al, 2003; Spitz and Furlong, 2012; Thomas and Buecker, 2023). How enhancers regulate transcription at great distances and select the genes that they regulate remains a mystery despite the over 40 years since their first discovery in viral genomes (Banerji et al, 1981).

Enhancers consist of reiterated binding sites for diverse sets of TFs that act combinatorially to ensure correct spatial and temporal control of gene expression (Spitz and Furlong, 2012; Whyte et al, 2013) (Fig. 1B). In addition to TFs, enhancers are known to recruit a variety of cofactors required for the regulation of transcription, such as the Mediator complex and RNAP II itself (Narita et al, 2021; Reiter et al, 2017; Soutourina, 2019; Whyte et al, 2013). The presence of these factors at the enhancer is believed to somehow translate into an  increase in the effective concentration of these factors in the vicinity of the promoter (Boija et al, 2018; Chen et al, 2014; Cho et al, 2018; Sabari et al, 2018; Whyte et al, 2013).

As enhancers are dispersed over the whole genome, how they select and regulate target gene expression has been extensively debated (Furlong and Levine, 2018; Galouzis and Furlong, 2022). In general, there are two modes of action by which an enhancer can act at a distance: DNA looping, where the enhancer forms a loop to interact with the promoter, or facilitated tracking, where the factors loaded onto the enhancer then track along DNA until they find the promoter (Furlong and Levine, 2018; Kane et al, 2022).

While the genome is clearly organized in 3D (Hsieh et al, 2022), there has been considerable debate about whether enhancers and promoters colocalize with the regulation of transcription. Recent studies that have attempted to image enhancer–promoter interactions suggest that transcriptional activity does not directly correlate with increased physical proximity between those elements (Alexander et al, 2019; Benabdallah et al, 2019). Moreover, single-molecule imaging of nascent transcription in *Drosophila* embryos showed that a single enhancer can simultaneously communicate with multiple promoters in opposite directions, suggesting that enhancer activity cannot be simply explained by DNA looping (Fukaya et al, 2016). How then do enhancers function from a great distance? One explanation that begins to reconcile these ideas is that they form aggregates, hubs, or factories. In these regions, the high concentration of TFs and auxiliary factors generates an environment that facilitates transcription (Tsai et al, 2017; Mir et al, 2018). One suggested biophysical model to explain the activity of these hubs would be the formation of a phase-separated region in which TFs diffuse slowly and are therefore retained in the vicinity of their regulatory element (Boija et al, 2018; Chong et al, 2022; Ji et al, 2024; Ma et al, 2021; Sabari et al, 2018; Stortz et al, 2024; Zamudio et al, 2019). The properties of phase-separated transcriptional hubs are poorly described, and many experiments are performed in vitro under nonphysiological conditions (Stortz et al, 2024; McSwiggen et al, 2019). Even though their functional role is controversial, transcription hubs have been observed in live cells, and their formation linked to the presence of IDRs in both EDs and cofactors, suggesting that IDRs play a role in compartmentalizing transcriptional regulators at enhancers (Boija et al, 2018; Ma et al, 2021; Patil et al, 2023; Sabari et al, 2018).

What enables an enhancer to stimulate compartmentalization or the recruitment of sufficient cofactors to regulate transcription at a distance? Unlike prokaryotic operons, enhancers are founded on combinatorial cooperativity, as they integrate the action of a wide range of TFs and signaling pathways into a single transcriptional output (Kim and Maniatis, 1997; Panne et al, 2007; Siersbæk et al, 2014; Zamudio et al, 2019). It is estimated that enhancers in higher eukaryotes comprise ~3–50 clusters of distinct TFBSs (Mirny, 2010), and reconstitution experiments in embryonic stem cells (ESCs) show that a minimum of ten TFBSs is required to drive enhancer activity (Singh et al, 2021). Moreover, a significant subset of enhancers takes the form of highly cooperative large “super enhancers” (SEs) that are themselves composed of multiple enhancer units (Whyte et al, 2013). The reiteration of these units both provides for robust changes in transcription but also provides multiple opportunities for combinatorial regulation (Chronis et al, 2017; Kim et al, 2023; Soufi et al, 2015). Even within the individual enhancer sequence, the orientation, affinity, and spacing of TFBS can be key for TFs to act in a combinatorial manner (Thanos and Maniatis, 1995; Georgakopoulos-Soares et al, 2023; Jindal and Farley, 2021). In these instances, a fixed motif grammar of tightly overlapping binding sites can produce the highly cooperative formation of a superstructure known as the enhanceosome, which is involved in viral induction of the Interferon-beta (IFN-β) enhancer (Panne et al, 2007). However, not all enhancer sequences are this rigid, and many do not require a fixed orientation of specific TFBSs. A more permissive model that exploits combinatorial TF activity to support both flexibility and synergy through protein-protein interactions allows for innovation in motif grammar and distinct cell type-specific responses (Singh et al, 2021; Arnosti and Kulkarni, 2005; Spitz and Furlong, 2012). These findings support the concept of a flexible level of cooperativity that relies less on specific TF–TF interactions, or the location of specific DNA sites, but rather on the holistic interface of a set of TFs with their targets and the combinatorial activities of their respective IDRs.

### Cooperativity and dimerization

Enhancers contain sets of reiterated elements that exploit the interaction among TFs and their shared interaction with coactivators and the transcription complex more generally. These interactions give rise to synergy or cooperativity that enables enhancers to overcome the threshold required for transcription activation and makes gene expression dependent on the combinatorial input (Carey 1998; Chronis et al, 2017; Junion et al, 2012; Panne et al, 2007; Siersbæk et al, 2014; Soufi et al, 2015). The principle behind cooperativity is that the affinity of the TF for DNA or its interaction with coactivators can be enhanced by the interaction of two TFs, or two TFs with a common target (Courey, 2001; Kerppola and Curran, 1991; Ptashne, 2006; Treisman et al, 1992). While this can be simply visualized at the level of cooperative binding, it can also be manifested at the level of transcriptional synergy (Kim and Maniatis, 1997) (Fig. 1B).

The simplest conceptual mechanism to explain combinatorial regulation is dimerization. Many TFs form hetero- or homodimers to produce distinct patterns of DNA recognition (Amoutzias et al, 2008). For example, bZIP domain proteins feature monomers that each recognize distinct DNA sequences (half sites), such that different dimeric combinations recognize distinct sites; the JUN and FOS heterodimer (AP-1) binds a TRE (“TGACTCA”), while heterodimers formed between JUN and proteins of the ATF/CREB family a CRE (“TGACGTCA”) (Hai and Curran, 1991). This combinatorial flexibility is essential for cell fate decisions, where different TF dimer combinations specify distinct lineages (Rodríguez-Martínez et al, 2017; Sekyrova et al, 2010; Tominaga et al, 2008).

More generally, one of the earliest paradigms for cooperativity is the switch-like behavior regulating the choice between lysis and lysogeny in phage lambda. When lambda C1 reaches a critical threshold concentration, it binds cooperatively to two adjacent operator sequences to promote lysogenic growth (Ptashne, 2004). Based on this paradigm, cooperativity is thought to be a ubiquitous feature of mammalian development. For example, in ESCs, their undifferentiated state is maintained by the combinatorial function of the TFs SOX2, OCT4, and NANOG, which bind together to a large set of regulatory elements to control their activity (Li and Belmonte, 2017; Singh et al, 2021; Whyte et al, 2013). SOX2 and OCT4 also cooperatively recognize composite sites to activate transcription synergistically (Yuan et al, 1995; Chen et al, 2014; Ambrosetti et al, 1997). In developmental contexts, cooperativity can be exploited to control both cell fate specification and the timing of these processes. For instance, OCT4 and SOX2 together constrain epithelial differentiation to enable tissue morphogenesis, and similarly, the interaction between NANOG and ESRRB enables plasticity during inner cell mass differentiation to guide specification of the blastocyst (Chandramohan et al, 2025; Knudsen et al, 2023). Altering the stoichiometry of these factors can then trigger commitment to differentiation.

Given the role of IDRs in multivalent interactions and DNA site recognition (Brodsky et al, 2020; Chen et al, 2022; Currie et al, 2017; Kumar et al, 2023; Ozkan et al, 2025; Tuttle et al, 2021), they are a strong candidate in facilitating cooperativity, particularly as they may mediate enhancer configurations or hubs (Boija et al, 2018; Zamudio et al, 2019; Zhao et al, 2023). At this level, combinatorial regulation becomes blurred and suggests that the correct biochemical configuration of a cooperative complex may not be essential. Studies in yeast point to this sort of principle, showing that combinatorial binding of TFs does not necessarily follow cooperativity and that only a small subset of TFs acts cooperatively (Jana Lang et al, 2024; Lupo et al, 2023).

### Transcription factor binding to chromatin

While TFs, a significant number of cofactors, and the transcriptional machinery can interact directly with DNA, large segments of DNA are not freely accessible in a cell, posing a challenge for transcription (Klemm et al, 2019). DNA is packaged into chromatin with the simplest unit being the nucleosome, each consisting of approximately 147 base pairs of DNA wrapped twice around a histone octamer composed of H2A, H2B, H3, and H4 (McGinty and Tan, 2015). This octamer can then be gated by linker histones such as H1 or H5, which bind to 10 bp of DNA at either side of the histone octamer (Allan et al, 1980; Li et al, 2024). The core histones all have a characteristic helical globular domain and a flexible amino-terminal tail that is the subject of post-translational modification. Modification of this tail region can influence how tightly wound the nucleosome is (Mariño-Ramírez et al, 2005). They also serve as a template for the binding of “readers”, which can in turn mediate levels of compaction, and they are introduced by “writers,” cofactors that can be recruited by TFs (Gillette and Hill, 2015). Accessible chromatin typically contains loosely packed nucleosomes that feature acetylated lysines, and various other PTMs in addition to nucleosome-depleted regions that can be detected using assays such as DNase hypersensitivity or ATAC-seq (Cockerill, 2011; Grandi et al, 2022; Otterstrom et al, 2019; Klemm et al, 2019).

Transcriptionally active chromatin regions are characterized by histone modifications that are associated with a relaxed chromatin state. Active enhancers are associated with H3K27ac and H3K4me1 (Creyghton et al, 2010), often also H3K4me3 (Pekowska et al, 2011), while promoters are associated with H3 and H4 acetylation (Wang et al, 2008) and H3K4me3 (Lauberth et al, 2013; Talbert et al, 2019). Within the gene body, regions of active transcription also contain high levels of acetylation (Martin et al, 2021). Regions of repressed chromatin feature H3K9me3 (Nicetto et al, 2019), H3K27me3 (Cai et al, 2021), and H2AK119ub1 (Endoh et al, 2012). Chromatin that is accessible and transcriptionally active is called euchromatin, whereas inactive chromatin is referred to as heterochromatin. The regulation of euchromatin and heterochromatin is highly dynamic, particularly during development and cell-state transitions (Gaspar-Maia et al, 2011; Ugarte et al, 2015; Zhu et al, 2013). Genes embedded within heterochromatin must be recognized and activated by TFs, but how do TFs access their targets? Certain TFs can recognize sequences in this context and have therefore been termed pioneer factors, as they find their targets in heterochromatin and recruit various chromatin modifiers to induce more accessible chromatin states that are amenable to transcription (Barral and Zaret, 2024).

Although pioneer factors are often described as a distinct class of transcription factors, this group is broad, and their activity can vary depending on the biological context (e.g., motif content, TF concentration, cell-type, and cofactor availability) (Freund et al, 2024; Gibson et al, 2024; King and Klose, 2017; Hansen and Cohen, 2022). While it is hard to explicitly define a pioneer factor, TFs that bind nucleosome-assembled DNA in vitro can be said to belong to this class, and there are clearly TFs that cannot do this (Cirillo et al, 2002; Zhu et al, 2018; Soufi et al, 2015). Moreover, in vitro experiments suggest that engaged TF binding to a nucleosome core can destabilize its association with DNA (Frederick et al, 2023; Soufi et al, 2015). Structural studies reveal that pioneer factors employ distinct binding strategies, often targeting nucleosome entry or exit sites (Barral and Zaret, 2024; Roberts et al, 2021). This interaction distorts DNA conformation, thereby promoting DNA unwrapping and nucleosome eviction (Mivelaz et al, 2020; Guan et al, 2023).

To achieve and maintain an open chromatin state, pioneer TFs frequently recruit chromatin remodelers such as switch/sucrose non-fermentable (SWI/SNF) and the NuRD complex (Gong et al, 2022; King and Klose, 2017; Matsui et al, 2024; Wolf et al, 2023). These remodelers reposition or remove nucleosomes, increasing DNA accessibility, enabling additional TF binding, and creating an environment permissive for transcriptional activation (Gong et al, 2022; King and Klose, 2017). Pioneer factor activity is particularly important in regulating cell states, to access compact chromatin and activate previously silent genes required for establishing a new cellular identity (Soufi et al, 2015; Liang et al, 2008; Matsui et al, 2024; Wang et al, 2015). While the recruitment of these remodelers depends on the ED contained in these pioneer factors, their capacity to recognize nucleosome-assembled DNA may also exploit activities outside of the DBD. In isolation, deletion mutants consisting solely of the DBD frequently fail to access their targets, implying that regions beyond the DBD could contribute to full pioneering activity (Gibson et al, 2024). IDRs have been implicated in guiding pioneer factor binding, facilitating the displacement of histone H1 (Ji et al, 2024; Frederick et al, 2023), and recruiting chromatin remodelers to promote chromatin opening (Zolotarev et al, 2024), suggesting that multivalency and their flexible properties could be required to localize and overcome nucleosome DNA barriers.

### Transcription factors supporting plasticity

While pioneer factors were named based on their capacity to search out their targets in chromatin, recent findings suggest that the activity of TFs can be uncoupled from transcription. They can act as pioneer factors by opening closed chromatin, creating transcriptional potential, or remaining after transcription has occurred, recording a cellular memory and supporting plasticity (Figs. 1B and 2B) (Barral and Zaret, 2024; Hamilton et al, 2019). In this way, TFs can compete with chromatin assembly after replication (Wenger et al, 2023), exploit the cell cycle or metabolic signals to configure the correct set of primed and decommissioned enhancers to promote high-fidelity differentiation (Wong et al, 2023), or remain bound at enhancers following transcription to enable potential dedifferentiation or regeneration (Hamilton et al, 2019; Linneberg-Agerholm et al, 2024).

Together, these activities of TFs, which are independent of transcription but ensure robust and high-fidelity transcriptional responses when cells are challenged with signaling, we term plasticity (Fig. 2B). For example, in response to FGF/ERK signaling, both in vivo at the blastocyst stage and in vitro, in ESCs, differentiation is highly reversible. This plasticity is maintained by TF occupancy at enhancers by factors like ESSRB, SOX2, and OCT4 (Hamilton et al, 2019; Linneberg-Agerholm et al, 2024). These enhancers appear maintained in a plastic, accessible state decorated by histone modifications associated with activation (H3K27ac and H3K4me1) and, to some extent, repression (H3K27me3) (Linneberg-Agerholm et al, 2024). A similar chromatin configuration is acquired in human ventral foregut stem cells as they proliferate and TFs like FOXA1 and HHEX maintain enhancers in a state prepared for later hepatic and pancreatic differentiation (Wong et al, 2023). Such a chromatin state can also be primed metabolically in ESCs, setting their enhancer states for future potential extra-embryonic as well as embryonic differentiation (Bone et al, 2025).

Taken together, these studies on plasticity suggest that TFs that contain canonical EDs can sit on an enhancer without stimulating transcription of a nearby gene; in these contexts, their IDRs presumably have a role in creating an environment conducive to gene transcription without stimulating mRNA production. What do these states constitute, and what is the switch that then promotes active transcription? One possible explanation would be that signal transduction is required to modify the IDR environment and shift a permissive transcriptional state into an active one (Fig, 2B).

## Transcriptional activation

Transcriptional activation involves regulation on many different levels and requires sequential and cooperative interplay of various factors at different stages, including chromatin accessibility, cofactor recruitment, complex assembly, DNA melting, RNAP II pause release, and elongation (Cramer, 2019). Below, we consider these steps and the regulatory opportunities they present (Fig. 1B).

Traditionally, TFs were thought to recognize specific elements via their DBD in enhancers and recruit cofactors to promote transcription. However, TF occupancy occurs in diverse contexts, can be influenced by IDRs or regulatory domains, and is not always linked to active transcription (Brodsky et al, 2020; Hamilton et al, 2019; Richter et al, 2022; Spitz and Furlong, 2012). What governs TF specificity and activity? Evidence suggests that both cofactor recruitment and PTMs mediated by signaling pathways play important roles (Galbraith et al, 2013; Hamilton et al, 2019; Narita et al, 2021; Reiter et al, 2017; Wang et al, 2009; Zamudio et al, 2019). For the sake of this discussion, cofactors can broadly be grouped into three categories: chromatin modifiers, GTFs, and the Mediator complex. Consequently, transcriptional output is not an inherent property of a TF alone but rather emerges from a complex combinatorial interplay involving distinct sets of cofactors.

### Chromatin modifiers

Transcription is regulated at multiple levels, starting with the action of enzymes that modify nucleosomes, individual histones and associated factors. Initially, for the transcriptional machinery to be recruited and to initiate transcription, DNA must be accessible (Skene et al, 2014; Fuda et al, 2015), and the first step in regulation is the preparation of the DNA template for recruitment of RNAP II and associated factors. While this process can be initiated in advance to create plasticity, it frequently involves pioneer factors acting at two levels. First, they can initiate chromatin decompaction by directly destabilizing the nucleosome at their binding site (Frederick et al, 2023; Soufi et al, 2015; Mivelaz et al, 2020). Second, they can recruit enzymes that either reposition nucleosomes or function as writers of histone modifications (Frederick et al, 2023; King and Klose, 2017; Miao et al, 2022).

Four main families of chromatin-remodeling complexes are directly responsible for ATP-dependent nucleosome rearrangements: The imitation switch (ISWI), SWI/SNF, chromodomain helicase DNA-binding (CHD), and INO80 (Clapier et al, 2017). These complexes all use ATP hydrolysis to fuel the translocation of DNA, rearrange nucleosomes, increase the spacing between nucleosomes, induce nucleosome sliding, or simply evict histones to create a nucleosome-free DNA (Clapier et al, 2017; Becker and Workman, 2013).

Another prominent mechanism by which chromatin accessibility is regulated is via the enzymatic modification of histone tails. The most common histone modifications are acetylation, phosphorylation, methylation, and ubiquitylation, which can be either associated with transcriptional activation or repression (Zhang et al, 2015). These marks act as dynamic recognition platforms for reader proteins (e.g., 14-3-3, HP1) (Dong et al, 2023; Zippo et al, 2009), chromatin-remodeling complexes (Sharma et al, 2021; Mansfield et al, 2011), and components of the PIC (Vermeulen et al, 2007; Lauberth et al, 2013), thereby influencing different steps in transcription activation.

Histone acetylation, particularly on lysine residues, is strongly correlated with transcriptional activity and is catalyzed by histone acetyltransferases (HATs) such as p300/CBP, GCN5, and MYST family enzymes (Calo and Wysocka, 2013). The removal of these modifications is typically associated with transcriptional repression and is carried out by histone deacetylases (HDACs) (Haberland et al, 2009). Active enhancers are typically decorated with H3K27ac, which is deposited by p300/CBP. p300/CBP was found to regulate the expression of highly active pluripotency genes in ESCs, associated with super enhancers, by stabilizing the PIC and promoting the recruitment of RNAP II (Narita et al, 2021). However, while p300/CBP activity is often essential, H3K27ac itself is not strictly required for enhancer activity (Zhang et al, 2020), indicating that p300/CBP also acts through mechanisms beyond histone acetylation. As p300/CBP acetylates a wide range of non-histone proteins (e.g., PIC components, Mediator, P-TEFb, and elongation complex components as well as TFs themselves), H3K27ac may be an effective marker of activity with the relevant acetylations elsewhere, highlighting the complexity of HAT-mediated regulation (Weinert et al, 2018). It also features IDR regions and can participate in condensate formation (Ma et al, 2021). However, this aspect of p300 is highly dynamic, as it has self-acetylating activity that inhibits condensate formation, and it can readily be mobilized in response to ERK signaling (Hamilton et al, 2019; Zhang et al, 2021).

Like acetylation, methylation of histones can also be dynamically regulated through the action of histone methyltransferases and histone demethylases, thereby modulating transcription outcome (Hyun et al, 2017). In mammalian cells, the SET1/MLL complexes deposit H3K4me3, a mark found at active promoters and associated with transcription initiation (Sze et al, 2020). H3K4me3 facilitates the recruitment of chromatin remodelers and PIC components such as TFIID, which supported a role in transcription initiation (Wang and Helin, 2025). However, acute depletion of H3K4me3 does not affect initiation, but instead impairs RNAP II promoter-proximal pause release as a result of a failure to recuit the Integrator subunit INTS11 (Wang et al, 2023). Elongation is often associated with H3K36me3, which is deposited across the actively transcribed gene body and is known for recruiting HDACs to suppress intragenic transcription initiation from alternative TSSs (Carrozza et al, 2005; Wei et al, 2020).

On the contrary, histone modifications like H3K9me2/3 and H3K27me3 mark transcriptional repression and heterochromatin. These marks promote a highly compact chromatin state to limit the accessibility of TFs and cofactors that induce transcription (Hyun et al, 2017). H3K9me2/3 specifically marks constitutive heterochromatin, which involves regions that are permanently condensed (e.g., centromeres, repetitive DNA) (Padeken et al, 2022). H3K9me2/3 is introduced by histone methyltransferases (HMTs), SUV39H1/2, SETDB1/2 and G9A/GLP. The introduction of this modification leads to the recruitment of heterochromatin protein 1, which promotes chromatin compaction (Hyun et al, 2017; Dong et al, 2023). Unlike constitutive heterochromatin, facultative heterochromatin can transition between active and repressed states and is characterized by H3K27me3. This modification is associated with gene-specific transcriptional repression and often emerges during development to silence lineage-specific genes (Zhu et al, 2013; Blackledge and Klose, 2021). H3K27me3 is deposited by the Polycomb repressive complex 2 (PRC2) and serves to recruit PRC1 to compact chromatin (Blackledge and Klose, 2021). PRC1 also ubiquitinates H2AK119 to promote repression (Endoh et al, 2012). Histone lysine demethylases (KDMs), such as UTX and JMJD3, can remove H3K27me3 marks, thereby facilitating chromatin opening and transcriptional activation. Their recruitment can be regulated by TFs and signaling (Dahle et al, 2010; Seenundun et al, 2010).

While there is some functional data discussed above on the association of specific histone PTMs and recruitment of factors (e.g., TFIID and PIC), most of the evidence for their activity is correlative. As we consider the recruitment of TFs via IDRs, the role of IDRs in multivalent interactions, their modification, and the potential alteration of histone tails, it is likely that these histone PTMs may contribute to an overall microenvironment that is conducive to TF access or transcription itself.

### Recruitment and initiation

Once the DNA template is free of nucleosomes, this does not mean transcription will occur. Transcription initiation involves three main steps: formation of the PIC to recruit RNAP II to the promoter, promoter melting to unwind DNA around the TSS, and promoter escape, where RNAP II disengages from most GTFs and begins synthesizing a short transcript of 10–20 nucleotides (Cramer, 2019). All three phases represent stages upon which TFs can act. Based on in vitro reconstitution of aspects of PIC assembly, TFs have been ascribed roles in promoting PIC assembly via interaction with different components (e.g., TFIIB and TFIID) (Liu et al, 2009; Chen and Pugh, 2021; Franklin et al, 1995; Coleman et al, 2017; Berkenstam et al, 1992; Ing et al, 1992; Baniahmad et al, 1993; MacDonald et al, 1995). In addition, TFs can promote promoter melting as they can directly bind and recruit TFIIH (Pearson and Greenblatt, 1997; Xiao et al, 1994), which acts via its XPB translocase module to unwind DNA during transcriptional initiation (Grünberg et al, 2012; Holstege et al, 1996).

A second factor involved in RNAP II initiation is the Mediator complex, that is both directly targeted by TFs, engaging with RNAP II during recruitment and disengaging during the initiation of transcription (Kornberg, 2005). The Mediator integrates information from TFs bound to enhancers and signaling pathways and conveys this information to the promoter (Fernkorn and Schröter, 2025; Galbraith et al, 2013; Hamilton et al, 2019; Soutourina, 2019; Wang et al, 2009). The core Mediator complex comprises head, middle, and tail and it reversibly associates with a kinase module that features CDK8 (Soutourina, 2018). Its tail module interacts with TFs, while its head engages RNAP II (Petrenko et al, 2016; Soutourina, 2019; Borggrefe and Yue, 2011; Plaschka et al, 2015), providing a means for signaling pathways and enhancer-bound TFs to communicate with RNAP II. For instance, the tail subunits MED23, 24, and 25, in addition to the central scaffold MED14, have all been linked to ERK-mediated enhancer regulation (Currie et al, 2017; Galbraith et al, 2013; Hamilton et al, 2019; Monté et al, 2025; Wang et al, 2009). These interactions are thought to involve multivalent contacts mediated by IDRs, which can be further tuned by PTMs (Currie et al, 2017; Datta et al, 2025; Tuttle et al, 2021; Warfield et al, 2014; Zamudio et al, 2019). Multivalent interactions between the Mediator and TFs facilitate the formation of dynamic transcriptional hubs at regulatory sites (Boija et al, 2018; Sabari et al, 2018; Zamudio et al, 2019; Cho et al, 2018). These hubs are highly attuned to signaling, which can modulate charge and thereby influence TF or cofactor incorporation (Zamudio et al, 2019; Weaver et al, 2022, Monteiro et al, 2026) (Fig. 2B).

### Promoter-proximal pausing

As recruited RNAP II frequently pauses at promoters, a well-established means to activate transcription is the release of promoter-proximally paused RNAP II into productive elongation (Adelman and Lis, 2012). Similar to initiation, TFs and signaling converge on this regulatory step (Ohe et al, 2022; Williams et al, 2015). As RNAP II is already loaded and initiated, this form of regulation can enable particularly rapid transcriptional responses and was originally described in *Drosophila* heat shock signaling (Rougvie and Lis, 1988; Schwartz et al, 2003).

Promoter-proximal pausing is established shortly after transcription initiation, when RNAP II has escaped the PIC, but has not yet entered productive elongation. During early transcription, the C-terminal domain of RNAP II (CTD), which is composed of tandem heptapeptide repeats, is phosphorylated at serine 5 by the CDK7 kinase of TFIIH. This modification promotes the release of RNAP II from the PIC and enables the transition into early elongation, by producing a short transcript of about 50 nucleotides (Chen et al, 2018; Schwartz et al, 2003). The phosphorylation of the CTD at serine 5 also causes the dissociation of the Mediator complex from the PIC, seperating the Mediator-RNAPII complex from the GTFs and allowing promoter escape (Wong et al, 2014). Subsequently, in early elongation, factors such as NELF and DSIF act on RNAP II to induce a state of ‘promoter-proximal pausing’, typically occurring 25-50 nucleotides downstream of the TSS (Core and Adelman, 2019). RNAP II can be released from pausing through the recruitment of positive transcription elongation factor β (P-TEFb) (Marshall and Price, 1992), a kinase that phosphorylates NELF and DSIF, culminating in NELF dissociation (Core and Adelman, 2019). Concurrently, P-TEFb phosphorylates the RNAP II C-terminal repeats at serine 2, a modification that marks the transition from early to productive elongation (Bowman and Kelly, 2014). Thereafter, RNAP II interacts with various elongation factors, which promote the synthesis of full-length pre-mRNA transcripts (Core and Adelman, 2019).

Promoter-proximal pausing of RNAP II enables rapid gene activation, a mechanism that is commonly used in signaling pathways where target genes must respond quickly to extracellular stimuli (Core and Adelman, 2019; Adelman et al, 2009; Williams et al, 2015; Liu et al, 2015). The MAPK signaling pathway, one of the most ubiquitous pathways in development and differentiation, activates the transcription of immediate early genes via pause release (Hazzalin and Mahadevan, 2002). The central nuclear kinase, ERK, phosphorylates NELF-A, leading to its disassociation (Ohe et al, 2022). Moreover, ERK can also induce Mediator recruitment to enhancers (Hamilton et al, 2019), and Mediator can promote pause release through its associated CDK8 module, which recruits P-TEFb (Donner et al, 2010). As a result, TF recruitment of Mediator to enhancers could establish a rapid response complex, where the local concentration of CDK8 can respond directly to signaling and contribute to pause release. As transcription can be regulated at multiple levels, promoting “partial activation” can be a means of supporting potential for later regulation or transcriptional plasticity.

## Conclusion

What guides TFs to their sites at enhancers? DBDs and IDRs clearly play a role, but what directs IDR localization? When signaling induces the recruitment of Mediator, p300, and PTMs, enhancers become highly dense regions of dynamic peptide motifs or a molecular “hub” or metaphorical molecular “mud” (Boija et al, 2018; Sabari et al, 2018; Zamudio et al, 2019; Cho et al, 2018). TFs are recruited into this hub both by specific sequences, and by the presence of transcription itself, as observed for the localization of factors such as SOX2 and ESRRB in response to ERK signaling (Hamilton et al, 2019). These TFs often leave non-consensus sites and appear in regions with the highest levels of induced transcription, again with no consensus site, contributing to a permissive environment that amplifies gene expression (Monteiro et al, 2026; Hamilton et al, 2019).

**Figure 3:**
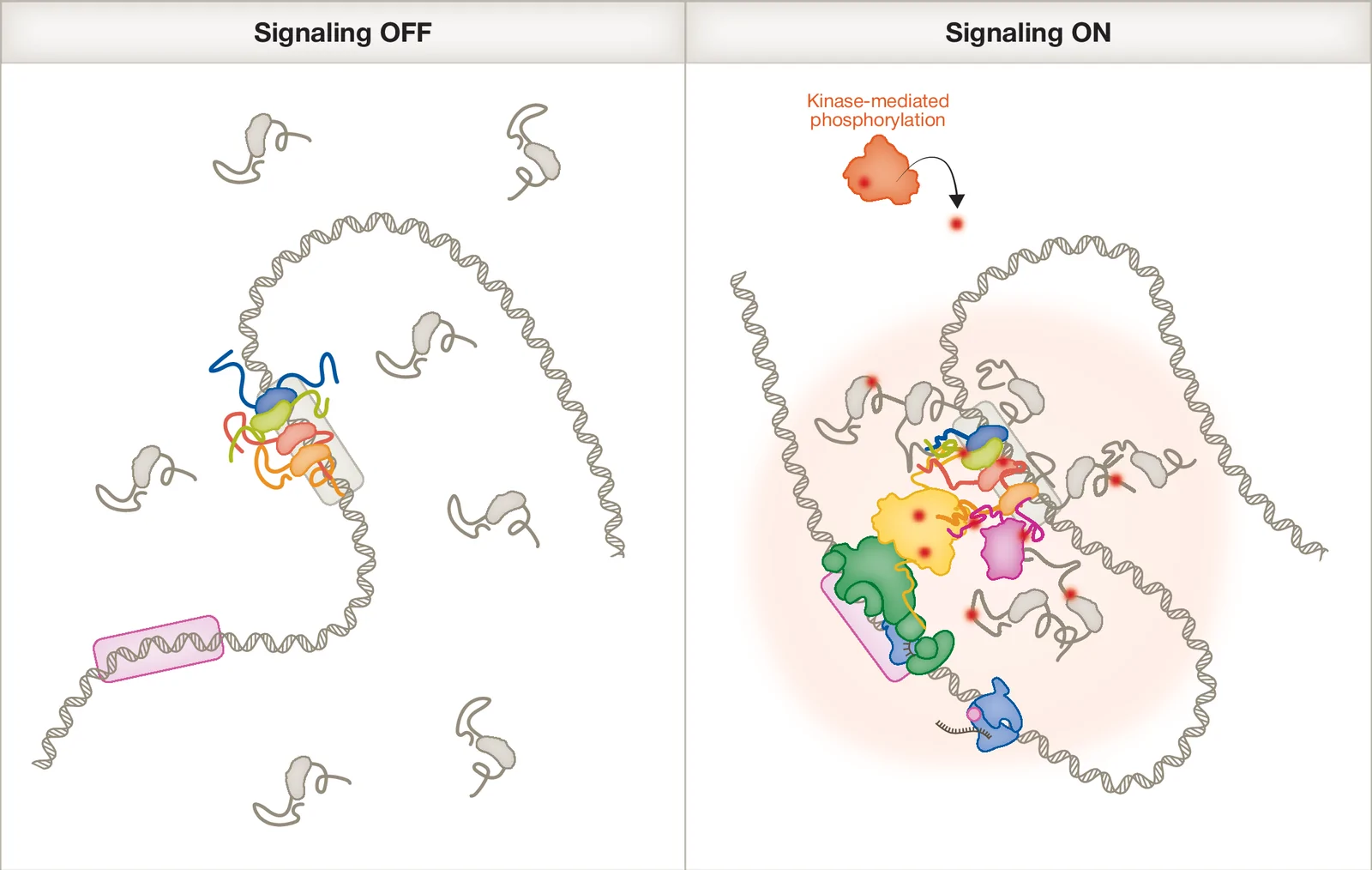
Transcriptional activation and signaling. Cartoon depicting the two roles we envision for TF activity. In the left panel, TFs act as competence or plasticity factors. They localize to high-affinity sites on their enhancer (grey box in the left panel) and ensure that a specific gene is licensed for future activation in response to signaling. In the right panel, transcription is depicted in response to signaling. Here, the TF IDRs are recruited to a region of already active transcription, where these factors are present at a high concentration to ensure productive transcription in a feed-forward mechanism. The red dots represent PTMs (such as phosphorylation) that occur in TFs, cofactors, and IDRs. On the right, the gray TFs are depicted as not interacting with DNA, as their recruitment appears DNA sequence independent, but it could also involve non-specific DNA binding.

Perhaps this suggests two modes of TF binding (Fig. 3): First, high-affinity interactions at consensus sites, priming enhancers for transcription. This could occur via the recruitment of specific chromatin-modifying enzymes (Wolf et al, 2023; Judd et al, 2021; Narita et al, 2021) or the generation of a modest level of eRNA (Mousavi et al, 2013; Saha et al, 2024), both of which would be compatible with enhanced cofactor binding and eventual transcription. These primed elements determine what a cell can become in differentiation or regeneration, rather than what it is. Second, during robust transcriptional responses driven by signaling and other TFs, additional TF molecules are recruited via their IDRs (Zamudio et al, 2019; Garcia et al, 2021; Zhao et al, 2023; Monteiro et al, 2026). In this mode, TFs act to directly facilitate high levels of mRNA production. Thus, TFs function both as competence factors and as active regulators of transcriptional output. The retention of TFs at sites of active transcription both enhances transcriptional responses and constrains TF distribution, perhaps explaining why pioneer factors only recognize a small proportion of their naturally occurring sites (Larson et al, 2021). As a result, TF properties and target recognition are encoded in complex patterns of high and low affinity DNA sites, or enhancer grammar, and the combinatorial output of different IDR sequences. Understanding how this complexity is manifested as discrete and specific biological switches is a major challenge for the future and will be the foundation for any approaches to engineer synthetic regulatory circuits (Box 1).

Box 1 In need of answers

1. How do signaling pathways regulate properties of local transcriptional microenvironments within chromatin to control specific transcriptional events?
2. Do IDRs within TFs encode a compositional grammar that regulates the specificity of transcriptional outcomes?
3. How do signaling-dependent PTMs within IDRs modulate their specific interactions with cofactors?
4. How do TF IDRs act combinatorially to produce apparent cooperativity or synergy at specific regulatory elements? How does signaling exploit this cooperativity in the activation of transcription?
5. Do IDR-mediated interactions contribute to transcriptional plasticity and cell-state transitions?

To address these questions, it will be important to move beyond tools that measure a population-averaged signal of TF binding, such as those obtained from ChIP-seq, CUT&RUN, etc. Instead, one would need to move towards locus-resolved and dynamic approaches. One strategy to obtain locus-specific information about a TF function is to combine single-particle tracking of TFs with fluorescently labeled genomic loci (e.g., TetO arrays and DNA-FISH). In this case, TetO arrays could be used to label regulatory elements that are permissive to signaling, and a Halo-tagged TF used to determine the influence of signaling on binding dynamics. In parallel, these loci could also be studied using proximity labeling proteomic approaches to characterize the composition and changes to it in the local environment.

Both approaches would enable context and locus-specific characterization of IDR function on complex assembly and stability. Systematic perturbations, including protein degradation and optogenetic modulation of protein expression, could be used to probe the role of specific factors. The function of individual domains and/or IDRs could then be probed by inducible rescue. Moreover, designing synthetic IDRs in this context could help in understanding whether there is a specific grammar that applies to these regions and regulates TF function in response to signaling.

If adapted to high throughput, such strategies could generate datasets that are suitable to train computational models that can predict transcriptional responses from TF/cofactor composition and IDR features.

Finally, these experimental frameworks could be applied to study transcriptional plasticity during cell-state transitions, helping to distinguish how TFs function in different contexts, as competence factors or as active drivers of transcription.

## Supplementary information

Peer Review File
