# Supplementary material for: A transcription factor by any other name - function beyond DNA binding and transactivation
Source: EMBO Rep. 2026 Sep 10;27(19):5429–48. doi: 10.1038/s44319-026-00893-3 (PMC13650123; doi:10.1038/s44319-026-00893-3)
Supplement: Supplementary file 1 [file 44319_2026_893_MOESM1_ESM.pdf]

EMBO reports

A transcription factor by any other name - function beyond DNA binding and transactivation

Christina Maria Schuh and Joshua Mark Brickman

Corresponding author(s): Joshua Brickman (joshua.brickman@sund.ku.dk)

**Review Timeline:**		
	Submission Date:	15th Dec 25
	Editorial Decision:	2nd Feb 26
	Revision Received:	12th Apr 26
	Accepted:	22nd Jul 26

Editor: Esther Schnapp

Transaction Report:

(Note: With the exception of the correction of typographical or spelling errors that could be a source of ambiguity, letters and reports are not edited. Depending on transfer agreements, referee reports obtained elsewhere may or may not be included in this compilation. Referee reports are anonymous unless the Referee chooses to sign their reports.)

Dear Josh,

Thank you for the submission of your Review EMBO reports. Below you will find the three sets of comments we have now received on your manuscript.

As you will see, all referees acknowledge that the review is new and interesting, which is great. Especially referees 1 and 3 also have several suggestions for how the review could be improved and I think all suggestions are good and I would like to ask you to address these.

If possible, it would be good to add one more figure to the review, as figures are usually very helpful to illustrate specific points or aspects of the article and often facilitate a rapid understanding of complex issues.
Please upload all figures as individual, high resolution figure files and add the legends as plain text (not in a box) to the end of the ms file.
I think we can use the figures as they are but if you prefer to involve a graphics designer, please let me know. It will take approximately 10 working days for the designer to redraw the figures, after we will have received the final ms file from you.

The current title might be too long, but I will find out what our publisher can offer.

Both authors need to have profiles in our online ms submission system. Please add a profile for the second author.

Please add up to 5 keywords to the ms file.

Please add a "Disclosure and Competing Interest Statement " to the ms file.

The REFERENCE FORMAT needs to be corrected: - et al needs to be used after 10 author names; DOIs should only be used for preprints and datasets that have not been published yet. Please use the EMBO reports reference style.

A Box called "IN NEED OF ANSWERS" is missing and needs to be added to the review. This Box should list open questions in the field, which can be accompanied by suggestions for how these questions could be experimentally addressed.

As for timing, would it be possible for you to submit the revised version by early March? If you anticipate a problem meeting this deadline, then please just let me know.

Thank you again for writing this review for us, and I am looking forward to seeing this published!

Best wishes,
Esther

Esther Schnapp, PhD
Senior Editor
EMBO reports

Referee #1:

This is an expansive review of transcription. Its strength is the clarity of writing, the depth at which it covers various topics related to cell state control, and the emphasis on recent papers. Despite mentioning in the abstract that the focus is on the consequences of transcription factor (TF) binding on cell state transitions, there is extensive material that seems unrelated to cell state transitions and the review would be improved by cutting back or out various sections. I appreciate how the authors take a neutral or critical view of various popular or fashionable aspects of the field and I have one or two more suggestions along those lines. Overall we are in favor of the review, which will be particularly useful to upper-level undergraduates, first level graduate students, and all investigators outside the field wanting to learn more about it. Details follow.

1. Given the stated goal of the review, two full pages on the basal transcription complex at the outset seems like a bit of a distraction. This section could be reduced greatly, though I would not lose one of its main messages- that TF and coregulator assembly at the promoter is driven by various DNA motifs.

2. To me, it seems phenomenological to discuss how TFs interact with a wider range of sequences in vivo than expected from their in vitro activity, without considering chromatin states first; as it has been shown (for example) that heterochromatin can block many TFs from binding their target sites. Regardless of that point, excluding yeast most TFs target sites in vivo that their DBDs have defined from in vitro studies with chromatin-free DNA.

3. IDR section, which is generally well done. However, I disagree with the discussion of the Brodsky 2020 paper which tried to conclude that non-DBD IDRs localize TFs to sites that their DBDs then bind. While IDRs can certainly influence which sites are chosen, I don't draw the same conclusion from the data in their paper. In their analysis of whether the DBD mutant, IDR-only Msn2 construct targets sites like wild type Msn2 protein, it does not enrich at promoters like the DBD does and more importantly, the authors didn't show how the IDR-only construct may bind across the genome; they just showed at 100 normal Msn2 sites. Maybe it binds everywhere and lacks specificity without a DBD. Maybe it's a yeast-specific phenomenon; my lab has not seen this at all, in mammalian cells. In any event, I feel that a closer reading of that paper is warranted rather than the acceptance of the authors claims- an example of (in my view) a more critical reading would be useful.

4. While covering IDRs generally well, to me the big question is how any level of specificity arises, as not all IDRs promote interactions with all other proteins with IDRs. What do the authors think about how that will be resolved?

5. The sections on Enhancers, cooperativity, dimerization are all nicely done, just longer than needed with all the reviews out there. The meat of the paper, and where the authors' particular insights with regard to cell fate control are more novel, occur in the sections on chromatin, plasticity, through to the end. My suggestion is to greatly cut back on the prior parts (preserving much of the IDR section) and focus on the authors distinctions about chromatin opening, plasticity, and signal responsiveness, all of which are key to cell state changes.

6. Where disease relevance is mentioned, include specific examples (which TFs have DBD mutations linked to congenital disorders in Dai et al., 2021 and Jeong & Bulyk et al., 2025). Right now these claims read as unsubstantiated because no examples are given.

7. For the discussion that a given effector domain can activate or repress, it would help to give specific examples and importantly the cell state/fate context in which this switches. Which is another way of saying how well was that work done? As examples of different effector domains on a single TF, DelRosso et al. found that NANOG has distinct regions mediating repression versus activation. Including how NANOG's bifunctional activity relates to fate transitions and/or growth factor context would link the review's thesis more clearly (for example, Heurtier, Navarro et al., Nat Comms 2019: in response to LIF, NANOG promotes self-renewal through activation; in the absence of LIF, maintains H3K27me3 at differentiation loci). Additionally, DelRosso et al. found that many ZFPs have repressive domain, this seems worth stating explicitly.

8. A concrete example that directly links cooperativity to fate transitions would help here. For instance: Chandramohan, Bedzhov et al., Dev Cell 2025 found that loss of Sox2 or Oct4 across blastocyst stages leads to ectopic expression of trophectoderm (TE) genes. This could also link nicely to naïve-to-primed transitions and signaling cues, and link to some of the studies cited in the section above.

Minor:
I don't think the acronym ED was defined at first use (effector domain or...).

Referee #2:

This is a comprehensive report by a leader in the transcription field. It raises the critical conceptual questions guiding the field, and discuss them in the context of both classical and new work. This will likely become a landmark reference. i strongly support publication.
only a small note the authors are free to ignore: the conclusion of (Mahendrawada et al., 2025) ("high throughput screens in yeast indicate that many TFs exhibit a bifunctional role that is context specific (Mahendrawada et al., 2025)") was challenged in a biorxiv paper (doi: https://doi.org/10.1101/2025.10.12.681120) based on reanalysis of the data

Referee #3:

Overall, I think this is a useful review on an important topic. The authors present a thorough survey of the literature on transcription factors that will be helpful to those unfamiliar with the field. They highlight the interesting idea that in addition to their role as effector domains, IDRs may serve a more general function as protein-protein interaction modules.
My major recommendation is that the authors dig more deeply into the specifics of experimental observations underlying the claims made by the studies that they cite. Rather than presenting the various (sometimes contradictory) models in the literature as fact, it would be helpful to describe the evidence more explicitly and explain some of the caveats and limitations of the experimental approaches involved. The review could be strengthened by addressing the following comments:
• It is worth noting that the order of assembly of the GTFs is based on in vitro experiments. This may or may not correspond precisely to what occurs in cells.
• A "DNA binding domain" may be substantially greater than 60 amino acids if you consider multiple modules, e.g., multiple Zn

fingers, to be part of the same DBD. It is worth clarifying this point.
• The authors seem to contradict themselves by saying that "For TFs to regulate transcription, they generally need to recognize their respective TF binding site (TFBS), a specific DNA sequence, which in eukaryotes is between 6-12 bps long(Georges et al., 2010)." But also: "[Some TF] IDRs can be shown to mediate promoter localization in the absence of their DBD." It would be helpful to clarify that there is currently a tension between these two opposing models of specificity.
• It is worth noting that different assays of chromatin binding-ChIP, CUT&RUN, ChEC, and single molecule tracking (SMT)-have distinct biases, limitations, and tradeoffs. Each assay employs an indirect readout (crosslinking, DNA cleavage, or molecular mobility) as a proxy for binding. ChIP, CUT&RUN, and ChEC provide locus-specific resolution but require killing cells by fixation or permeabilization. SMT operates in live cells, but it lacks the genomic resolution of the other methods.
• Related to the previous point: Rather than just saying that "localization of Msn2 or Yap1 to promoters was shown to be IDR dependent", specify that this conclusion is based on ChEC-seq, and explain how this method works. Some have called the validity of this assay into question: https://www.biorxiv.org/content/10.1101/2021.02.04.429774v1.
• "Given their nature and the importance of charge in mediating their interactions with other factors" - unclear what "their nature" means.
• "These findings support the concept of a flexible level of cooperativity that relies less on direct TF-TF interactions" - Unclear what "direct" means in this context. Are cooperative interactions between TF IDRs not "direct"?
• "For example, the IDRs of the TFs OCT4 and GCN4 or the cofactors MED1 and BRD4 have been shown to support the formation of condensates in the nucleus (Boija et al., 2018)." - It would be worth unpacking a bit further what has actually been shown experimentally. What assays were used, and what are their caveats?
• "hypoxia-inducible factors HIF-1α and HIF-2α that bind identical sites in vitro, but in vivo have a distinct IDR-dependent binding profile (Y. Chen et al., 2022)" - unclear what "binding profile" means. This study showed a slight effect of IDRs in CUT&RUN but a much more dramatic effect in single-molecule tracking in live cells.
• "Similarly, live-cell imaging revealed that these IDRs help increase the time TFs stay bound and increase their local concentration, working together with the DBDs to find and remain at target sites and ultimately activate transcription (Abidi et al., 2025)." - Not quite an accurate synopsis of this paper. This manuscript does not report residence time measurements or effects on transcription. The main point was that IDRs mediate interactions between TFs at physiological concentrations, independent of DBD specificity, facilitating cooperative binding to chromatin. The authors reasoned that such cooperative interactions likely contribute to the binding specificity of TFs.
• "CTCF and cohesin [...] were found to be key in providing a physical link between enhancers and promoters" - This is a controversial claim, which recent evidence has called into question. Loop extrusion by cohesin decreases the average distance between enhancers and promoters within TADs (Lee et al., 2025 PMID 40966347), as in the extreme example of Shh discussed in subsequent paragraph. It is also true that there are special cases in which enhancers and promoters are present near CTCF loop anchors (Canzio et al., 2019, PMID 30955885). However, this seems to be the exception rather than the rule. It is not, in general, thought that CTCF and cohesin play a direct role in bridging most enhancers and promoters. This is indicated, for instance, by the persistence of E-P loops detected by MicroC following CTCF and cohesin depletion (Hsieh et al., 2022, PMID 36471071). CTCF/cohesin-dependent loops generally do not coincide with enhancers and promoters.
• "the formation of a phase-separated region in which TFs diffuse very rapidly" - This doesn't make physical sense, because the formation of a condensed phase typically slows diffusion. See, for example, Chong et al., 2022, PMID 35483357.
• "Even though their functional role is controversial, transcription hubs have been observed in live cells, and their formation was linked to the presence of IDRs of EDs and cofactors" - It is worth critically evaluating these observations. Many reports of visible "hubs" or "condensates" have involved fixation and immunofluorescence and overexpression of tagged proteins. Many published images also show obvious thresholding, with pixels below a certain intensity set to black, although this manipulation is seldom explained (for examples where it is disclosed see Guo et al. PMID 31391587 and Fig. S6 of Cho et al., PMID 29930094). Thresholding an under-labeled immunofluorescence image will make it look more punctate than the actual spatial distribution of the protein.
• "H3K27ac itself is not strictly required for enhancer activity (T. Zhang et al., 2020), indicating that p300/CBP also acts through mechanisms beyond histone acetylation." - This observation indicates that p300/CBP acts through mechanisms beyond acetylation of H3K27. However, it is important to note that histones are acetylated at multiple residues. The emphasis on H3K27Ac may be in part a historical accident due to the availability of good anti-H3K27Ac antibodies.
• "It can also promote RNP II promoter-proximal pause release through the recruitment of the Integrator complex (Lauberth et al., 2013; H. Wang et al., 2023)." - The Lauberth reference is about TFIID and doesn't seem to have anything to do with Integrator.
• "what compels the IDRs to localize?" - Consider whether this is the right way of framing the question. Might a better question be what IDRs interact with and how these interactions contribute to TF localization?
• P. 25: What is a "mud"? I've never heard this term.
• Fig. 2: What does it mean for TFs to be "absorbed in a region of competence"? Is there a way of describing more precisely what you mean? What does the pink circle represent, and why aren't any of the gray TFs with PTMs binding DNA? If you increase the effective local concentration of a DNA-binding protein via protein-protein interactions, won't that promote its binding to DNA?
Minor:
• I was momentarily confused by the abbreviation "ED". Perhaps make the section title "Effector domains (EDs)"
• "While the transcriptional machinery assembles for mRNA generation promoters" - maybe "at promoters to initiate transcription"?
• P. 22 "RNP II" - should be "RNAP II"

- "deposited by the Polycomb Repressive Complex 2 (PRC2) and stands in a crosstalk with PRC1" - not clear what "stands in a crosstalk" means. Consider rephrasing
- P. 25: Should have "The" at the beginning of the sentence before "MAPK signaling pathway"

Response to Reviewers Comments: Schuh and Brickman 2026

Referee #1:

This is an expansive review of transcription. Its strength is the clarity of writing, the depth at which it covers various topics related to cell state control, and the emphasis on recent papers. Despite mentioning in the abstract that the focus is on the consequences of transcription factor (TF) binding on cell state transitions, there is extensive material that seems unrelated to cell state transitions and the review would be improved by cutting back or out various sections. I appreciate how the authors take a neutral or critical view of various popular or fashionable aspects of the field and I have one or two more suggestions along those lines. Overall, we are in favor of the review, which will be particularly useful to upper-level undergraduates, first level graduate students, and all investigators outside the field wanting to learn more about it. Details follow.

1. Given the stated goal of the review, two full pages on the basal transcription complex at the outset seems like a bit of a distraction. This section could be reduced greatly, though I would not lose one of its main messages- that TF and coregulator assembly at the promoter is driven by various DNA motifs.

We agree with the reviewer, and we have cut the section in half.

2. To me, it seems phenomenological to discuss how TFs interact with a wider range of sequences in vivo than expected from their in vitro activity, without considering chromatin states first, as it has been shown (for example) that heterochromatin can block many TFs from binding their target sites. Regardless of that point, excluding yeast, most TFs target sites in vivo that their DBDs have defined from in vitro studies with chromatin-free DNA.

We thank the reviewer for this comment, and we have addressed by these different points:

- *We have added text on chromatin, and that it can influence TF binding, adding a selective layer in target choice (p.7): "*As DNA *in vivo* is packed into chromatin, nucleosomes or higher-order packing can block TF binding, limiting their access to the full spectrum of DNA sites within the genome (Grand et al., 2024). Consistently, TF binding is enriched in regions of open chromatin and excluded from densely packed nucleosomes in heterochromatin (Becker et al., 2016; Guertin & Lis, 2013; Lupien et al., 2008). While this selective binding could reflect chromatin access, even TFs that can recognize their sites in chromatin bind only

a small fraction of their full complement of high-affinity sites (King & Klose, 2017; Reiter et al., 2017; Slattery et al., 2011)."

- *We have kept this section short as we discuss chromatin extensively later in the review.*

3. IDR section, which is generally well done. However, I disagree with the discussion of the Brodsky 2020 paper, which tried to conclude that non-DBD IDRs localize TFs to sites that their DBDs then bind. While IDRs can certainly influence which sites are chosen, I don't draw the same conclusion from the data in their paper. In their analysis of whether the DBD mutant, IDR-only Msn2 construct targets sites like wild type Msn2 protein, it does not enrich at promoters like the DBD does, and more importantly, the authors didn't show how the IDR-only construct may bind across the genome; they just showed at 100 normal Msn2 sites. Maybe it binds everywhere and lacks specificity without a DBD. Maybe it's a yeast-specific phenomenon; my lab has not seen this at all, in mammalian cells. In any event, I feel that a closer reading of that paper is warranted rather than the acceptance of the authors claims- an example of (in my view) a more critical reading would be useful.

- *We agree with the reviewer, and we have acknowledged that this work on IDRs was done in yeast and have increased the nuance in this paragraph (p.7, line 168, 175) and also (p.11, line 283).*
- *We explicitly discuss that it is the top promoters and a phenomenon so far only found in yeast.*

4. While covering IDRs generally well, to me, the big question is how any level of specificity arises, as not all IDRs promote interactions with all other proteins with IDRs. What do the authors think about how that will be resolved? Interaction with specific cofactors or with DNA? Or both. Interesting point

- *We have addressed this point by adding a discussion of additional factors that the IDR interacts with to facilitate TF binding and localization (page 11, line 293)*

5. The sections on Enhancers, cooperativity, dimerization are all nicely done, just longer than needed with all the reviews out there. The meat of the paper, and where the authors' particular insights with regard to cell fate control are more novel, occur in the sections on chromatin, plasticity, through to the end. My suggestion is to greatly cut back on the prior parts (preserving much of the IDR section) and focus on the authors

distinctions about chromatin opening, plasticity, and signal responsiveness, all of which are key to cell state changes.

We thank the reviewer for this comment. We have edited the enhancer section and removed the discussion of loop extrusion, CTCF and cohesin as they are not relevant to the discussion of cooperativity.

We have also added a discussion of developmental timing (see response to 8) (p.15, line 421).

6. Where disease relevance is mentioned, include specific examples (which TFs have DBD mutations linked to congenital disorders in Dai et al., 2021 and Jeong & Bulyk et al., 2025). Right now, these claims read as unsubstantiated because no examples are given.

We thank the reviewer for this comment, and we have taken out this part.

7. For the discussion that a given effector domain can activate or repress, it would help to give specific examples and importantly the cell state/fate context in which this switches. Which is another way of saying how well was that work done? As examples of different effector domains on a single TF, DelRosso et al. found that NANOG has distinct regions mediating repression versus activation. Including how NANOG's bifunctional activity relates to fate transitions and/or growth factor context would link the review's thesis more clearly (for example, Heurtier, Navarro et al., Nat Comms 2019: in response to LIF, NANOG promotes self-renewal through activation; in the absence of LIF, maintains H3K27me3 at differentiation loci). Additionally, DelRosso et al. found that many ZFPs have repressive domain, this seems worth stating explicitly.

- *We thank the reviewer for this comment, and we believe this is relevant to the role TFs play in plasticity (p.15, line 421).*
- *We have added a sentence on TF behavior in stem cells as both activators and repressors (p.9, line 233) and added the references suggested.*

8. A concrete example that directly links cooperativity to fate transitions would help here. For instance, Chandramohan, Bedzhov et al., Dev Cell 2025 found that loss of Sox2 or Oct4 across blastocyst stages leads to ectopic expression of trophectoderm (TE) genes. This could also link nicely to naïve-to-primed transitions and signaling cues, and link to some of the studies cited in the section above.

- *We thank the reviewer for this comment, and we have added a discussion of cooperativity, plasticity and differentiation in developmental timing (p.15, line 421)*

Minor:
I don't think the acronym ED was defined at first use (effector domain or...).

We have introduced it now in the header of the section on Effector domains

Referee #2:

This is a comprehensive report by a leader in the transcription field. It raises the critical conceptual questions guiding the field, and discuss them in the context of both classical and new work. This will likely become a landmark reference. i strongly support publication.
only a small note the authors are free to ignore: the conclusion of (Mahendrawada et al., 2025) ("high throughput screens in yeast indicate that many TFs exhibit a bifunctional role that is context specific (Mahendrawada et al., 2025)") was challenged in a biorxiv paper (doi: https://doi.org/10.1101/2025.10.12.681120) based on reanalysis of the data

- *We thank the reviewer for this comment, and we have added this reference to our Effector Domain section (p. 9, line 231).*

Referee #3:

Overall, I think this is a useful review on an important topic. The authors present a thorough survey of the literature on transcription factors that will be helpful to those unfamiliar with the field. They highlight the interesting idea that, in addition to their role as effector domains, IDRs may serve a more general function as protein-protein interaction modules.
My major recommendation is that the authors dig more deeply into the specifics of experimental observations underlying the claims made by the studies that they cite. Rather than presenting the various (sometimes contradictory) models in the literature as fact, it would be helpful to describe the evidence more explicitly and explain some of the caveats and limitations of the experimental approaches involved. The review could be strengthened by addressing the following comments:

• It is worth noting that the order of assembly of the GTFs is based on in vitro experiments. This may or may not correspond precisely to what occurs in cells.

- *We have added the following sentence to the introductory paragraph for the GTFs, "The assembly of the PIC is based on in vitro reconstitution experiments and the extent to which these fractions exist as independent entities in vivo is unclear." (p.4, line 82).*

• A "DNA binding domain" may be substantially greater than 60 amino acids if you consider multiple modules, e.g., multiple Zn fingers, to be part of the same DBD. It is worth clarifying this point.

- *We thank the reviewer for this comment. We agree, and we have removed this statement for clarity.*

• The authors seem to contradict themselves by saying that "For TFs to regulate transcription, they generally need to recognize their respective TF binding site (TFBS), a specific DNA sequence, which in eukaryotes is between 6-12 bps long(Georges et al., 2010)." But also: "[Some TF] IDRs can be shown to mediate promoter localization in the absence of their DBD." It would be helpful to clarify that there is currently a tension between these two opposing models of specificity.

- *We disagree, as the next sentence in this paragraph discusses the multiple layers of interaction.*

• It is worth noting that different assays of chromatin binding-ChIP, CUT&RUN, ChEC, and single-molecule tracking (SMT)-have distinct biases, limitations, and tradeoffs. Each assay employs an indirect readout (crosslinking, DNA cleavage, or molecular mobility) as a proxy for binding. ChIP, CUT&RUN, and ChEC provide locus-specific resolution but require killing cells by fixation or permeabilization. SMT operates in live cells, but it lacks the genomic resolution of the other methods.

- This is true, but we feel this sort of discussion is beyond the scope of this review. We would be happy to add this discussion, should the reviewer and editor think it necessary.

• Related to the previous point: Rather than just saying that "localization of Msn2 or Yap1 to promoters was shown to be IDR dependent", specify that this conclusion is based on ChEC-seq, and explain how this method works. Some have called the validity of this assay into question: https://www.biorxiv.org/content/10.1101/2021.02.04.429774v1.

- *We thank the reviewer for this comment and have added a sentence to the IDR section to create more nuance, including the caveats of ChEC-seq (p7., line 171) and (p.11, line 286)*

• "Given their nature and the importance of charge in mediating their interactions with other factors" - unclear what "their nature" means.

- *We are referring to disorder and have clarified it in the text. (p.9, line 237)*

• "These findings support the concept of a flexible level of cooperativity that relies less on direct TF-TF interactions" - Unclear what "direct" means in this context. Are cooperative interactions between TF IDRs not "direct"

- *We thank the reviewer for this comment, and we have changed the word "direct" to "specific" and have tried to clarify. Our idea is that all interactions in these complexes contribute to their overall stability producing an exponential enhancement to the different disassociation constants (p14, line 389): "*These findings support the concept of a flexible level of cooperativity that relies less on specific TF-TF interactions, or the location of specific DNA sites, but rather on the holistic interface of a set of TFs with their targets and the combinatorial activities of their respective IDRs in enhancing the overall stability of a multimeric complex"

• "For example, the IDRs of the TFs OCT4 and GCN4 or the cofactors MED1 and BRD4

have been shown to support the formation of condensates in the nucleus (Boija et al., 2018)." - It would be worth unpacking a bit further what has actually been shown experimentally. What assays were used, and what are their caveats?

While we believe listing all the caveats of all assays would require too much space in this review, we have added a sentence to address the reviewer's point (p. 10, line 273)

• "hypoxia-inducible factors HIF-1α and HIF-2α that bind identical sites in vitro but in vivo have a distinct IDR-dependent binding profile (Y. Chen et al., 2022)" - unclear what "binding profile" means. This study showed a slight effect of IDRs in CUT&RUN but a much more dramatic effect in single-molecule tracking in live cells.

*We thank the reviewer for the comment. We have clarified our statement: "*The ability of IDRs to influence TF binding is not unique to yeast proteins; similar findings have been reported for human hypoxia-inducible factors HIF-1α and HIF-2α that bind identical sites *in vitro*, but *in vivo,* they bind distinct target sites based on their IDRs (Y. Chen et al., 2022)."

• "Similarly, live-cell imaging revealed that these IDRs help increase the time TFs stay bound and increase their local concentration, working together with the DBDs to find and remain at target sites and ultimately activate transcription (Abidi et al., 2025)." - Not quite an accurate synopsis of this paper. This manuscript does not report residence time measurements or effects on transcription. The main point was that IDRs mediate interactions between TFs at physiological concentrations, independent of DBD specificity, facilitating cooperative binding to chromatin. The authors reasoned that such cooperative interactions likely contribute to the binding specificity of TFs.

- *We thank the reviewer for the comment, and we have corrected our statement (p. 11, line 296).*
- *We currently do not mention anything about either transcription or TF binding, so we apologize if we confused the reviewer.*
- *We have nuanced our discussion of IDRs and DNA binding specificity to include a role for the transcription complex to facilitate TF recruitment by IDRs as opposed to IDR interaction with DNA (also see response to reviewer 1, comment 4)*

• "CTCF and cohesin [...] were found to be key in providing a physical link between enhancers and promoters" - This is a controversial claim, which recent evidence has called into question. Loop extrusion by cohesin decreases the average distance between enhancers and promoters within TADs (Lee et al., 2025 PMID 40966347), as

in the extreme example of Shh discussed in the subsequent paragraph. It is also true that there are special cases in which enhancers and promoters are present near CTCF loop anchors (Canzio et al., 2019, PMID 30955885). However, this seems to be the exception rather than the rule. It is not, in general, thought that CTCF and cohesin play a direct role in bridging most enhancers and promoters. This is indicated, for instance, by the persistence of E-P loops detected by Micro-C following CTCF and cohesin depletion (Hsieh et al., 2022, PMID 36471071). CTCF/cohesin-dependent loops generally do not coincide with enhancers and promoters.

- *We thank the reviewer for this comment. We have cut the enhancer section, and we have taken out the part about enhancer-promoter looping as it appears to extensive for this section (also in response to reviewer 1, comment 8).*

• "the formation of a phase-separated region in which TFs diffuse very rapidly" - This doesn't make physical sense, because the formation of a condensed phase typically slows diffusion. See, for example, Chong et al., 2022, PMID 35483357.

- *We thank the reviewer for the comment. We apologize for the mistake, and we have corrected it (p. 13, line 356).*

• "Even though their functional role is controversial, transcription hubs have been observed in live cells, and their formation was linked to the presence of IDRs of EDs and cofactors" - It is worth critically evaluating these observations. Many reports of visible "hubs" or "condensates" have involved fixation and immunofluorescence and overexpression of tagged proteins. Many published images also show obvious thresholding, with pixels below a certain intensity set to black, although this manipulation is seldom explained (for examples where it is disclosed, see Guo et al. PMID 31391587 and Fig. S6 of Cho et al., PMID 29930094). Thresholding an under-labeled immunofluorescence image will make it look more punctate than the actual spatial distribution of the protein.

We thank the reviewer, and we have added a sentence on the different techniques used to study condensate formation, pointing out that their evidence is still far from conclusive (p.10, line 273).

While we agree with the reviewer, we want to get across different ideas and feel we could lose track of the general principles if we go into the caveats of each technique. However, please let us know if there is still an issue.

• "H3K27ac itself is not strictly required for enhancer activity (T. Zhang et al., 2020), indicating that p300/CBP also acts through mechanisms beyond histone acetylation." - This observation indicates that p300/CBP acts through mechanisms beyond acetylation

of H3K27. However, it is important to note that histones are acetylated at multiple residues. The emphasis on H3K27Ac may be in part a historical accident due to the availability of good anti-H3K27Ac antibodies.

- *We agree with the reviewer, and we have added a sentence about the serendipity of H3K27ac, being a marker rather than a functional modification (p.21, line 596).*

• "It can also promote RNP II promoter-proximal pause release through the recruitment of the Integrator complex (Lauberth et al., 2013; H. Wang et al., 2023)." - The Lauberth reference is about TFIID and doesn't seem to have anything to do with Integrator.

- *We apologize for the mistake, and we have updated the references.*

• "what compels the IDRs to localize?" - Consider whether this is the right way of framing the question. Might a better question be what IDRs interact with and how these interactions contribute to TF localization?

- *We thank the reviewer for the comment. We have rephrased the sentence: "DBDs and IDRs clearly play a role, but what directs IDR localization?" (p.25, line 782)*

• P. 25: What is a "mud"? I've never heard this term.

- *We thank the reviewer for the comment. It was our attempt of a bit of a colorful, literary reference to condensates. We have added the word metaphorical, and we would like to keep the expression: "*When signaling induces the recruitment of Mediator, p300, and PTMs, enhancers become highly dense regions of dynamic peptide motifs or a molecular "hub" or metaphorical molecular "mud" (Boija et al., 2018; Cho et al., 2018; Sabari et al., 2018; Zamudio et al., 2019)" (p.25, line 782).

• Fig. 2: What does it mean for TFs to be "absorbed in a region of competence"? Is there a way of describing more precisely what you mean? What does the pink circle represent, and why aren't any of the gray TFs with PTMs binding DNA? If you increase the effective local concentration of a DNA-binding protein via protein-protein interactions, won't that promote its binding to DNA?

- *We thank the reviewer for this comment. We have addressed this by better describing what we mean by region of competence. The grey TFs are not binding*

DNA, as we don't have any evidence that they are recruited to a specific site. They may be nonspecifically associated with DNA, and we have mentioned it now in the figure legend. The pink circle shows PTMs, and we have clarified that in this figure, we depict phosphorylation by ERK signaling.

Minor:

• I was momentarily confused by the abbreviation "ED". Perhaps make the section title "Effector domains (EDs)"

We thank the reviewer for the comment, and we have added ED to the section title

• "While the transcriptional machinery assembles for mRNA generation promoters" - maybe "at promoters to initiate transcription"?

We thank the reviewer for this comment, and we have changed it.

• P. 22 "RNP II" - should be "RNAP II"

We have corrected that.

• "deposited by the Polycomb Repressive Complex 2 (PRC2) and stands in a crosstalk with PRC1" - not clear what "stands in a crosstalk" means. Consider rephrasing

We thank the reviewer for the comment. We have clarified it: "H3K27me3 is deposited by the Polycomb Repressive Complex 2 (PRC2) and serves to recruit PRC1, which ubiquitinates H2AK119 to promote a compacted state (Endoh et al., 2012; X. Liu & Liu, 2022)."

• P. 25: Should have "The" at the beginning of the sentence before "MAPK signaling pathway."

We have corrected that.

1st Revision - Editorial Decision **22nd Jul 2026**

Prof. Joshua Brickman
University of Copenhagen
The Novo Nordisk Foundation Center for Stem Cell Medicine (reNEW)
University of Copenhagen
Blegdamsvej 3B
Copenhagen 2200
Denmark

Dear Josh,

I am pleased to inform you that your Review has been accepted for publication in EMBO reports. Your manuscript will be processed for publication by EMBO Press. It will be copy edited and you will receive page proofs prior to publication. Please note that you will be contacted by Springer Nature Author Services to complete licensing information.

If you have any questions, please do not hesitate to contact the Editorial Office. Thank you for your contribution to EMBO Reports.

Best,
Esther

Esther Schnapp, PhD
Senior Editor
EMBO reports

>>> Please note that it is EMBO Reports policy for the transcript of the editorial process (containing referee reports and your response letter) to be published as an online supplement to each paper. If you do NOT want this, you will need to inform the Editorial Office via email immediately. More information is available here: https://link.springer.com/partners/embo-press/editorial-policies#Peer%20review
